# Supplementary material for: Neurocognitive and mental health outcomes and association with quality of life among adults living with HIV: a cross-sectional focus on a low-literacy population from coastal Kenya
Source: BMJ Open. 2018 Sep 17;8(9):e023914. doi: 10.1136/bmjopen-2018-023914 (PMC6144406; doi:10.1136/bmjopen-2018-023914)
Supplement: Author's manuscript [file bmjopen-2018-023914.draft_revisions.pdf]

BMJ Open

BMJ Open is committed to open peer review. As part of this commitment we make the peer review history of every article we publish publicly available.

When an article is published we post the peer reviewers' comments and the authors' responses online. We also post the versions of the paper that were used during peer review. These are the versions that the peer review comments apply to.

The versions of the paper that follow are the versions that were submitted during the peer review process. They are not the versions of record or the final published versions. They should not be cited or distributed as the published version of this manuscript.

BMJ Open is an open access journal and the full, final, typeset and author-corrected version of record of the manuscript is available on our site with no access controls, subscription charges or pay-per-view fees (http://bmjopen.bmj.com).

If you have any questions on BMJ Open's open peer review process please email info.bmjopen@bmj.com

BMJ Open

Neurocognitive and mental health outcomes and association with quality of life among adults living with HIV: A cross-sectional focus on a low-literacy population from Coastal Kenya

Journal:	*BMJ Open*
Manuscript ID	bmjopen-2018-023914
Article Type:	Research
Date Submitted by the Author:	17-May-2018
Complete List of Authors:	Nyongesa, Moses; Centre for Geographic Medicine Research Coast, NeuroAssessment<br>Mwangala, Patrick ; Centre for Geographic Medicine Research Coast<br>Mwangi, Paul; Centre for Geographic Medicine Research Coast<br>Kombe, Martha; Centre for Geographic Medicine Research Coast<br>Newton, Charles; KEMRI-Wellcome Trust Research Programme, Centre for Geographic Medicine Research -Coast, Clinical Research<br>Abubakar, Amina A.; Centre for Geographic Medicine Research Coast
Keywords:	HIV & AIDS < INFECTIOUS DISEASES, Neurocognitive performance, MENTAL HEALTH, QUALITY OF LIFE, LITERACY, ADULTS

SCHOLARONE™ Manuscripts

For peer review only

Neurocognitive and mental health outcomes and association with quality of life among adults living with HIV: A cross-sectional focus on a low-literacy population from Coastal Kenya

Moses K. Nyongesa[1*], Patrick N. Mwangala[1], Paul Mwangi[1], Martha Kombe[1], Charles R. Newton[1, 2, 3], Amina Abubakar[1, 2, 3]

1 Neuroassessment Group, KEMRI-Wellcome Trust Research Programme, Center for Geographic Medicine Research (Coast), Kilifi, Kenya

2 Department of Psychiatry, University of Oxford, Oxford, United Kingdom

3 Department of Public Health, Pwani University, Kilifi, Kenya

 Corresponding author. E-mail address: Mkachama@kemri-wellcome.org, KEMRI-Wellcome Trust Research Programme, Center for Geographic Medicine Research (Coast), PO Box 230 (80108) Kilifi, Kenya.

Target Journal: BMJ Open

Word Count: 3693

For peer review only

Abstract

Objectives: Our aim was to compare the neurocognitive performance and mental health outcome of adults living with HIV on antiretroviral therapy with that of community controls; all of low-literacy. Furthermore, we also wanted to explore the relationship of these outcomes with quality of life among adults living with HIV.

Study design: This was a descriptive cross-sectional study.

Setting: The study was conducted in Kilifi County, a region located at the Kenyan Coast.

Participants: The participants consisted of a consecutive sample of 84 adults living with HIV and 83 randomly selected community controls all with ≤8 years of schooling. All participants were assessed for non-verbal intelligence, verbal working memory and executive functioning. The Major Depression Inventory (MDI) and a quality of life measure (RAND SF-36) were also administered.

Results: Using analysis of covariance (ANCOVA), we found no statistically significant group differences between adults living with HIV and community controls in all the neurocognitive tests except for a marginal difference in the non-verbal intelligence test ($F\ (1, 158) = 3.83,\ p=0.05$). However, depressive scores of adults living with HIV were significantly higher than those of controls ($F\ (1, 158) = 11.56,\ p<0.01$). Also, quality of life scores of adults living with HIV were significantly lower than those of controls ($F\ (1, 158) = 4.62,\ p=0.03$). For the HIV-infected group, results from multivariable linear regression analysis showed that increasing depressive scores were significantly associated with poorer quality of life ($\beta=-1.17$, *95% CI -1.55, -0.80*; $p<0.01$).

Conclusion: Our findings suggest that adults of low literacy levels living with HIV and on antiretroviral medication at the Kenyan Coast do not have significant cognitive deficits compared to their uninfected counterparts. However, their mental health, compared to that of HIV uninfected adults, remains poorer and their quality of life may deteriorate when HIV and depressive symptoms co-occur.

Keywords: HIV/AIDs, neurocognitive performance, mental health, quality of life, literacy, adults, Kenya

For peer review only - http://bmjopen.bmj.com/site/about/guidelines.xhtml

Strengths and limitations of this study

- This is one of the few studies simultaneously exploring cognitive, mental health and quality of life outcomes among HIV-infected sample from sub Saharan Africa.
- We also focus specifically on a low-literacy sample
- The key limitation is the cross-sectional nature of our study design, therefore, we cannot ascertain any causal relationship

For peer review only

For peer review only - http://bmjopen.bmj.com/site/about/guidelines.xhtml

1.0 Introduction

The introduction of highly active antiretroviral therapy (HAART) – more than three decades now – has resulted to significant declines in HIV/AIDs related morbidity and mortality [1]. An increase in life expectancy among individuals living with HIV and on HAART has also been observed in both low [2, 3] and high-resourced [4] settings. For HIV patients who are optimally treated with antiretroviral medication, their life expectancy may approach that of HIV uninfected population [5]. Despite this achievement in longevity, neurocognitive disturbance and psychological problems remain as important health issues of concern among people living with HIV/AIDs (PLWH) [6, 7].

Neurocognitive impairment (NCI) is a common complication following HIV infection [8, 9], even with the advent of HAART [10]. The NCI associated with HIV, also known as HIV-associated neurocognitive disorder (HAND), arises when HIV infected monocytes and macrophages infiltrate the brain by crossing the blood-brain barrier, to infect and activate microglia causing inflammation of the central nervous system and progressive cognitive dysfunction (from involvement of the cortical and subcortical regions) [9, 11]. In sub-Saharan Africa (SSA), where there is a substantial burden of HIV infection compared to other regions of the world, prevalence estimates for HAND as high as 42% (pre-HAART) and 30% (post-HAART) have been reported [12]. Some of the cognitive deficits described among PLWH, through neuropsychological testing, include impairment in attention/concentration, processing speed and executive functioning, as well as problems with motor functioning, learning and memory [13-15]. For PLWH on HAART and with well-controlled viremia, mild NCI is much more common than severe cognitive impairment i.e. HIV-associated dementia [10, 16]. There are significant clinical and functional consequences of having even mild NCI including: increased risk of mortality [17]; poor medication adherence [18]; decreased quality of life [19]; and disruptions to everyday functioning [20].

Mental disorders are also common among PLWH, depression being the most prevalent [21, 22]. A review of the mental health of adults living with HIV in Africa [21] reported estimates for depressive symptoms of over 30% and as high as 64% from different reviewed studies. Furthermore, major depressive disorder has been found to occur almost twice as frequent among PLWH compared to the HIV-uninfected subjects [23].

For peer review only

For peer review only - http://bmjopen.bmj.com/site/about/guidelines.xhtml

There are many reasons as to why PLWH may experience mental disorders frequently, particularly depressive symptoms. Firstly, is the difficulty of living with a chronic, life-threatening illness. This adjustment difficulty – presenting as psychological distress – may occur after receiving HIV positive diagnosis; when HIV symptoms begin to develop; at the start of lifelong antiretroviral medication; and also in terminal care [24]. Secondly, the direct effects of HIV virus on the central nervous system [25], may lead to behavioural changes resulting into symptoms of depression. Thirdly, such psychological disturbance may also be because of HIV-related stigma [26]. PLWH with comorbid mental illnesses may present with poor quality of life [22] and non-adherence to antiretroviral medication [27]. Non-adherence may lead to faster disease progression or increased HIV/AIDs related mortality.

Cognitive and mental health outcomes of PLWH and their relationship with quality of life have gained increasing attention recently, with most work being conducted and reported from highly resourced countries, but limited (although increasing) reports from SSA, where the burden of HIV/AIDS is substantial. To add to the body of research evidence from SSA, we evaluated the neurocognitive test performance and mental health of adults living with HIV on antiretroviral medication compared to community controls, specifically focusing on a low-literacy population at the Kenyan Coast. Less education among PLWH has been associated with an increased risk for symptomatic HAND [28, 29]. We also set out to explore the relationship between each of these outcomes with quality of life among adults living with HIV.

2.0 Methods

2.1 Study site:

This study was undertaken at the Centre for Geographic Medicine Research-Coast (CGMR-C), Kilifi County, Kenya between November 2016 and March 2017. The Centre has a Neuro-assessment unit equipped for assessing a wide range of neurocognitive and neuropsychiatric outcomes in children, adolescents and adults living with chronic illnesses such as HIV and epilepsy. Interviews and tests are carried out by trained and experienced research assistants. In Kilifi County, the estimated overall adult HIV prevalence is 4.5% with prevalence being higher among females (6.4%) than males (2.7%) [30].

2.2 Study design:

For peer review only - http://bmjopen.bmj.com/site/about/guidelines.xhtml

For peer review only

This was a cross-sectional study aimed at investigating neurocognitive functioning, mental health and their association with quality of life in an adult sample. This study was embedded in a larger study whose aim was to validate a tablet-based android application (App) – the NeuroScreen, Swahili version – screening for neurocognitive impairment among chronically ill adults in Kilifi. In the NeuroScreen study, only participants with ≤8 years of schooling (up to primary level of education in the Kenyan education system) were included with the assumption that if the App works with the less literate individuals in Kilifi, then it should work well with those who are better educated.

2.3 Sample size estimation:

In this current study, the sample size (n=167) included 84 participants living with HIV and 83 community controls. This sample size was calculated to detect differences in cognitive outcomes between adults living with HIV and controls with 90% power using previously reported effect sizes [31, 32].

2.4 Recruitment and criteria for inclusion:

Participants living with HIV: Adults living with HIV were recruited from the Comprehensive Care and Research Clinic (CCRC), an HIV specialised clinic at the Kilifi County Hospital. Selection and booking for assessment date was done through consecutive sampling of clients on their arrival at the clinic. Participants were eligible for inclusion in the study if they: i) were 20 to 50 years old; ii) had a confirmed diagnosis of HIV; iii) had no more than primary level of education (an inclusion criterion for the larger study validating the NeuroScreen); iv) could understand and communicate in Kiswahili (the official national language of Kenya) with ease; v) provided informed consent; and vi) had no obvious symptoms of acute illness on the day of assessment.

Community Controls: community controls were identified through the Kilifi Health and Demographic Surveillance System (KHDSS) by random selection and later approached at home and requested to take part in the study. Details about the KHDSS can be found elsewhere [33]. Those who consented to taking part in the study were referred to CGMR-C for assessment if they met the eligibility criteria as above, except that of having an HIV diagnosis. These participants had to self-report a seronegative test result in the last year. We did not test for HIV among these controls, and we acknowledge that in randomly selecting them, about 3 or 4 out of the 84 may have been HIV-infected considering the adult HIV prevalence in Kilifi County, Kenya [30]. However, through a detailed clinical examination

For peer review only - http://bmjopen.bmj.com/site/about/guidelines.xhtml

(see study procedures below), we only included participants having no symptoms of an acute illness. Using this criterion, we expected very few cases of asymptomatic HIV-infection among our controls that is unlikely to invalidate our findings.

Figure 1 below is a flowchart showing the recruitment process for both adults living with HIV and community controls in line with the STROBE guidelines for reporting observational studies [34].

Figure 1: Flowchart of participant recruitment

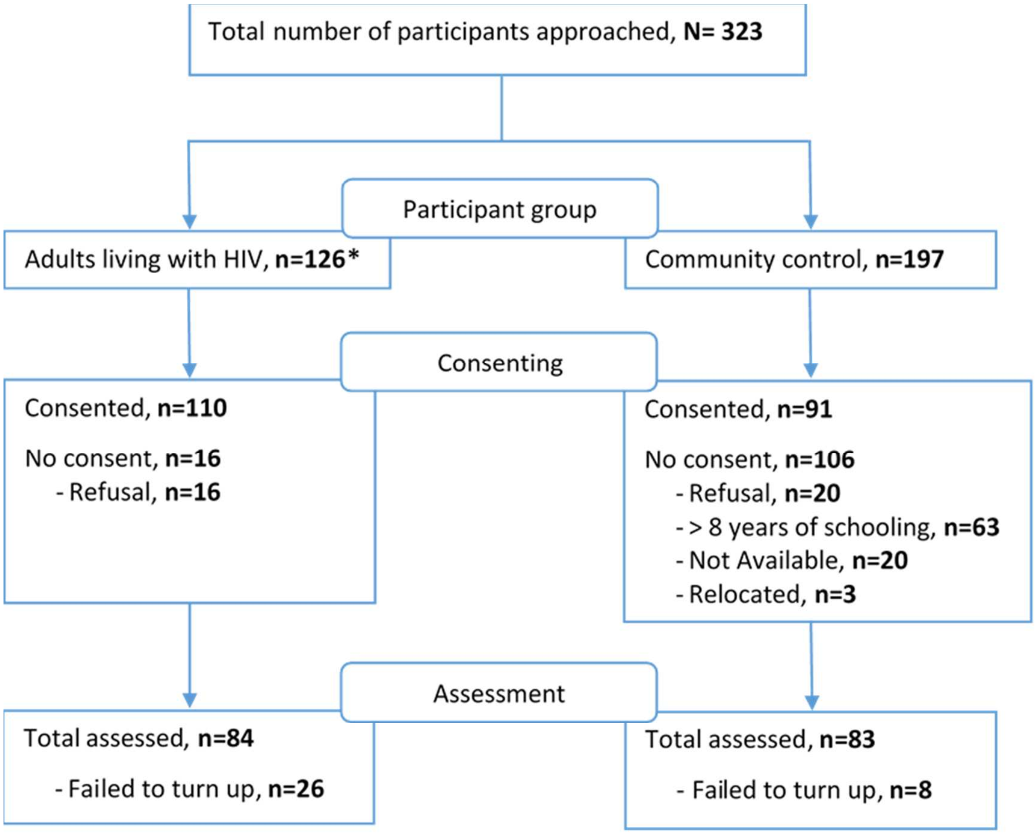

N/B: * Identified as having ≤8 years of schooling

2.5 Study Procedures:

Every consented participant underwent a detailed medical history and symptomatology clinical examination. For controls, other than ruling out symptoms of an obvious acute illness for participation, symptomatologic evaluation was also done to rule out the possibilities of HIV infection. A set of questionnaires were then administered to capture information about sociodemographic and economic background, mental health and quality of life. Cognitive

assessments were paper and pencil and computer tablet-based. Data collection was conducted by a trained team of research assistants.

2.6 Measures:

The measures listed below were all administered to both adults living with HIV and community controls. For both groups, all measures were administered in the same order, by the same assessor (who was blinded) and under the same administration environment.

Social demographic and asset index forms: These were used to collect information on participant's age, sex, marital status, level of education, religion and socioeconomic status. The asset index has been reliably used to assess socioeconomic status over time in studies conducted in this setting [35, 36]. The asset index items ask for ownership of disposable assets by participants (or their family) such as radio, television, bicycle or motorbike et cetera.

Major Depression Inventory (MDI): This is a self-report measure of depressive symptoms that examines moods experienced by an individual during the past 2 weeks. It consists of 12 items, although only 10 items are scored since for items 8 (a and b) and 10 (a and b) only an alternative with the highest score is considered. Items are scored on a 6-point Likert scale from 0 (at no time) to 5 (all the time) with the total score ranging from 0 to 50 [37]. The MDI can be used both as a diagnostic instrument for major depression according to the Diagnostic and Statistical Manual for Mental Disorders, version four (DSM-IV) and as a depression rating scale. As a rating scale, MDI total score of 20-24 is indicative of mild depressive symptoms, a score of 25-29 is indicative of moderate depressive symptoms whereas a score $\geq$ 30 indicates severe depressive symptoms [38]. This measure, adapted to Swahili, yielded reliable psychometric properties among young adults in Kilifi [39].

Measures of cognitive performance: In this study, three tests were administered to assess cognitive functioning among study participants i.e. the Raven's Standard Progressive Matrices (RSPM) [40], Contingency Naming Test (CNT) [41] and the Backward Digit span test [42]. RSPM is a non-verbal standardized test of intelligence quotient (IQ) comprising of five series with 12 items without limit in execution time. The CNT, initially developed to assess aspects of executive functioning such as processing speed and response inhibition in children, has shown good psychometric reliability when extended to the adult population [43]. In the CNT test, a participant is required to name the colour or shape of a series of stimuli, as fast and accurately as possible, according to different rules. The Backward Digit

For peer review only - http://bmjopen.bmj.com/site/about/guidelines.xhtml

Span test assesses verbal working memory [42]. A list of random numbers is read aloud to the participant who is then required to recall the items in a reverse order.

The RAND 36-Item Health Survey 1.0 (RAND SF-36): This is the most widely used generic measure of health-related quality of life. It assesses 8 health concepts: physical functioning, role limitations due to physical health problems, role limitations due to emotional problems, social functioning, emotional wellbeing, energy/fatigue, pain, and general health perceptions [44]. Scoring the tool involves various steps. Firstly, pre-coded numeric values are converted to percentages using a predetermined scoring key. Secondly, items in the same scale are summed and then averaged to give the 8 different sub-scale scores. Lastly, to generate a total score, all item scores are summed and then averaged. Higher scores (as percentage) define a more favourable state of health. RAND SF-36 has been found to be reliable in SSA [45]. For this study, the English questionnaire was forward translated into Kiswahili by two independent translators. Back translation into English was then undertaken by two other independent back translators (unaware of the original version). The final version was obtained following a harmonization process by a team of experts and incorporation of changes resulting from a cognitive interview process and pretesting procedures. In the current study, Cronbach's alpha for this measure was excellent at 0.93.

2.7 Ethical consideration:

This study was approved by the Kenya Medical Research Institute Scientific and Ethics Research Unit (Ref no: KEMRI/SERU/CGMR-C/030/3187). Permission to work in the comprehensive care clinic was also obtained from the Ministry of Health, Kilifi County government. Voluntary and written informed consent was obtained from all study participants after a detailed explanation of the study had been made to them.

2.8 Patient and Public Involvement

Patients were not involved in the design and conduct of the study

2.9 Statistical analyses:

All analyses were carried out on STATA (version 14.0) statistical software package [46]. Frequencies, percentages, mean or median were used to describe sample characteristics. Chi-square tests (with Fisher's exact test for continuity correction where appropriate) and independent student's t-test (or Wilcoxon rank sum test where appropriate) were used to compare group differences between categorical and continuous variables respectively.

For peer review only - http://bmjopen.bmj.com/site/about/guidelines.xhtml

Analysis of covariance (ANCOVA) was used to control for covariates of interest when looking at group differences in neurocognitive test performance, depressive and quality of life scores between adults living with HIV and community controls. We checked for ANCOVA's homogeneity of regression slope assumption using a method suggested by Johnson [47]. In an analysis involving only the HIV-infected group, we used correlational and multivariable linear regression analyses to explore potential associations between neurocognitive test performance, depressive scores and quality of life scores. All tests in our analyses were considered statistically significant at p <0.05.

3.0 Results.

Sample characteristics: Table 1 below describes sample characteristics by HIV status (i.e. adults living with HIV versus controls). The mean age of the 167 study participants who took part in the study was *36.95* (standard deviation [SD]= 8.65). Majority of the study participants were: females (*71.3%*); married (*63.9%*); and Christians (*81.1%*). Less than a quarter of study participants (*16.4%)* did not have any formal education. As shown in Table 1, adults living with HIV were significantly different from controls in all sociodemographic characteristics except for religion (*p=0.27*) and socioeconomic status (*p=0.92*). Nutritional status of the study participants (as indicated by body mass index and mid upper-arm circumference) and the clinical information of adults living with HIV are also presented in Table 1. We also present observations with missing values.

Table 1: Sample characteristics of adult participants living with HIV and community controls (n=167) along with clinical characteristics of participants living with HIV.

Sample Characteristics	Total sample	HIV infected n=84	Controls n=83	P value
	n (%) or mean(SD) or Median (IQR)			
**Age (in years; mean [SD])**	37.0 (8.7)	40.1 (6.4)	33.8 (9.5)	**<0.01[Ψ]**
**Sex**				
Female	119 (71.3)	66 (78.6)	53 (63.9)	**0.04**
Male	48 (28.7)	18 (21.4)	30 (36.1)	
**Marital status OM=1**				
Never married	27 (16.3)	8 (9.5)	19 (23.2)	**<0.01***
Married	106 (63.9)	47 (56.0)	59 (72.0)	
Separated/Divorced/Widowed	33 (19.9)	29 (34.5)	4 (4.9)	
**Education OM=2**				
None	27 (16.4)	20 (24.1)	7 (8.5)	**<0.01**
Primary incomplete	73 (44.2)	47 (56.6)	26 (31.7)	
Primary complete	65 (39.4)	16 (19.3)	49 (59.8)	
**Religion OM=8**				
Christian	129 (81.1)	64 (77.1)	65 (85.5)	0.27*

For peer review only

For peer review only - http://bmjopen.bmj.com/site/about/guidelines.xhtml

Muslim	14 (8.8)	10 (12.1)	4 (5.3)	
Other e.g. traditional	16 (10.1)	9 (10.8)	7 (9.2)	
**SES Asset Index (median [IQR])**	2.0 (1.0, 3.0)	1.0 (1.0, 3.0)	2.0 (1.0, 2.0)	0.92$^{\Omega}$
**BMI (median [IQR])**	22.4 (20.2, 26.2)	22.4 (19.0, 26.7)	22.3 (20.5, 25.9)	0.67$^{\Omega}$
**MUAC (median [IQR])**	26.9 (25.0, 29.3)	26.7 (24.2, 29.0)	27.1 (25.2, 29.4)	0.33$^{\Omega}$
**Severity of depressive symptoms**				
No depressive symptom	147 (88.0)	69 (82.1)	78 (94.0)	0.06*
Mild depressive symptoms	9 (5.4)	8 (9.5)	1 (1.2)	
Moderate depressive symptoms	6 (3.6)	4 (4.8)	2 (2.4)	
Severe depressive symptoms	5 (3.0)	3 (3.6)	2 (2.4)	
**HIV-related clinical characteristics**				
**HIV disease staging OM=6**				
Stage 1	NA	38 (48.7)	NA	NA
Stage 2	NA	27 (34.6)	NA	NA
Stage 3	NA	13 (16.7)	NA	NA
**Number of ARV medication OM=7**				
Two	NA	56 (72.7)	NA	NA
Three	NA	21 (27.3)	NA	NA
**Duration on ARV**				
< 1 year	NA	4 (5.1)	NA	NA
1-5 years	NA	29 (37.2)	NA	NA
6-10 years	NA	36 (46.2)	NA	NA
> 10 years	NA	9 (11.5)	NA	NA

N/B: Percentages are exclusive of missing values. For observations with missing values, the number of missing values is indicated in the table as **OM** (observations with missing values). P-values are based on chi-squared statistic except for: ***** - chi-square based on Fisher's exact test; **Ω** - based on Mann-Whitney U-test; **Ψ** – based on independent t-test; **SD** – standard deviation; **IQR** – inter quartile range; **SES** – socioeconomic status; **CNT** – Contingency Naming Test; **QoL** -Quality of life; **BMI** – Body Mass Index; **MUAC** – Mid upper-arm circumference **ARV** – antiretroviral; **NA** – Not Applicable

3.1 Neurocognitive performance, mental health and quality of life of adults living with HIV compared to community controls

Neurocognitive performance: in the crude analysis, adults living with HIV compared to controls had significantly lower mean non-verbal IQ scores as measured by the RSPM (*p<0.01; Table 2*) and verbal working memory scores i.e. lower mean total correct scores (*p=0.01; Table 2*) and mean score of highest level reached *(p<0.01; Table 2*) of the backward digit span test. However, no significant difference was observed between adults living with HIV and controls in executive functioning as assessed by CNT total error scores (*p=0.32; Table 2*). Using ANCOVA adjusting for sex, age, level of education and socioeconomic index as covariates, there was no significant differences between adults living with HIV and community controls for all neurocognitive test scores except non-verbal IQ scores where a marginal difference was observed, $F\ (1,\ 158) = 3.83$, p=0.05, with a small effect size (*0.29; Table 2*).

For peer review only - http://bmjopen.bmj.com/site/about/guidelines.xhtml

Mental health: Twenty (12.0%) of study participants met the criteria for mild to severe depressive symptoms (15 being PLWH and 5 being community controls) as measured by the MDI. Adults living with HIV had significantly higher mean depressive scores than controls (*p<0.01; Table 2*) in the crude analysis. ANCOVA controlling for sex, age, education and socioeconomic status (*Table 2*) still found significant differences between adults living with HIV and community controls, *F (1, 158) = 11.56, P<0.01*.

Quality of life: in the crude analysis, adults living with HIV compared to controls had significantly lower mean quality of life scores as measured by the RAND SF-36 (*p<0.01; Table 2*). Controlling for sex, age, education and socioeconomic status in ANCOVA (*Table 2*), there was still a statistically significant difference between adults living with HIV and community controls, *F (1, 158) = 4.62, p=0.03*.

Table 2: Unadjusted and adjusted analyses for neurocognitive test performance, mental health and quality of life outcomes comparing adults living with HIV and community controls

	Unadjusted analysis			Adjusted analysis*					
**Outcome**	**HIV score**	**Control score**	**p - value**	**HIV score**	**Control score**	**F - statistic**	**p - value**	**eta-squared (%)**	**ES**
**Neurocognitive test performance**									
CNT total errors	2.82 (0.92)	2.68 (0.85)	0.32	2.89 (0.12)	2.79 (0.12)	0.38	0.54	6.5	0.09
Ravens total score	16.82 (5.39)	19.81 (7.28)	**<0.01**	16.72 (0.82)	18.92 (0.83)	3.83	**0.05**	14.6	0.29
Digit Span -total correct score	5.06 (2.81)	6.41 (3.32)	**0.01**	4.85 (0.39)	5.42 (0.41)	1.05	0.31	18.7	0.16
Digit Span - highest level reached	2.19 (1.26)	2.90 (1.44)	**<0.01**	2.06 (0.17)	2.47 (0.18)	3.05	0.08	21.9	0.27
**Mental health**									
Depressive scores	11.80 (8.38)	7.96 (7.32)	**<0.01**	12.23 (1.03)	7.46 (1.04)	11.56	**<0.01**	11.71	0.50
**Quality of life**									
QoL total score	70.38 (17.18)	78.07 (14.72)	**<0.01**	72.56 (2.12)	78.78 (2.14)	4.62	**0.03**	9.64	0.32

NB: All scores presented as mean (SD), unadjusted analysis; and mean (SE), adjusted analysis; **ES** – Cohen's d effect size
***ANCOVA** adjusted for sex, age, level of education and socioeconomic index

3.2 Correlation of neurocognitive, depressive and quality of life scores in the HIV-infected group

There was no statistically significant correlation between any of the neurocognitive scores and quality of life scores i.e. total correct score (*r=-0.05, p= 0.63*), score of the highest level reached (*r=-0.01, p=0.96*), CNT's total error scores (*r=0.08, p=0.45*) or Ravens total score (*rho=-0.05, p=0.68*). However, correlation analysis revealed a significant correlation between depressive and quality of life scores (*rho=-0.67; p<0.01*). The reported results in the subsequent sections will therefore focus on findings from analyses that were carried out based on the observed significant correlation between depressive and quality of life scores.

For peer review only - http://bmjopen.bmj.com/site/about/guidelines.xhtml

3.3 Association between depressive symptoms and quality of life in the HIV-infected group

Exploring further the observed significant correlation between depressive and quality of life scores among adults living with HIV, a one-unit increase in depressive score was associated with 1.2 reduction in quality of life scores in the univariate linear regression analyses (*$p<0.01$; Table 3*). Adjusting for age, sex, number of and duration on antiretroviral medication and HIV disease staging, there was still strong evidence of an association between elevated depressive scores and poorer quality of life (*$\beta=-1.17$, 95% CI -1.55, -0.80; $p<0.01$*; *Table 3*).

Table 3: Univariate and multivariable linear regression analyses for the association between elevated depressive scores (exposure) and poorer quality of life (outcome) among adults living with HIV in Coastal Kenya, n=72

	Univariate analysis			Multivariable analysis		
**Exposure variable**	**unadjusted β Coefficient (95% CI)**	**p-value**	**R-squared (%)**	**adjusted β Coefficient* (95% CI)**	**p-value**	**R-squared (%)**
Depressive scores	-1.17 (-1.53, -0.80)	**<0.01**	36.70	-1.17 (-1.55, -0.80)	**<0.01**	45.19
**Adjusted for:**						
Age				-0.20 (-0.77, 0.37)	0.48	
Sex (male vs female)				7.02 (-1.18, 15.22)	0.09	
Number of ARV medication (3 vs 2)				-4.49 (-12.32, 3.33)	0.26	
HIV staging						
Stage 2 vs 1				-2.63 (-10.04, 4.77)	0.48	
Stage 3 vs 1				-8.60 (-18.12, 0.91)	0.08	
Duration on ARV						
1-5 years vs <1 year				7.83 (-6.63, 22.29)	0.28	
6-10 years vs <1 year				9.29 (-5.16, 23.73)	0.20	
>10 years vs <1 year				11.28 (-4.87, 27.43)	0.17	

NB: * Linear regression adjusted coefficient for quality of life; **ARV** – antiretroviral

4.0 Discussion

In this study, we were interested in evaluating neurocognitive and mental health outcomes of adults living with HIV compared to community controls, specifically focusing on a low-

For peer review only - http://bmjopen.bmj.com/site/about/guidelines.xhtml

literacy sample from the Kenyan Coast. We also wanted to explore the relationship between each of these outcomes and quality of life among the HIV-infected adult group.

4.1 Neurocognitive test performance

In summary, we did not observe main effect of HIV infection on most of the neurocognitive tests except that of non-verbal intelligence when comparing adults living with HIV and community controls all with lower educational background. For non-verbal intelligence, the adjusted group difference was marginally significant with a small effect size (0.3). We think that this marginal difference may be due to impaired premorbid intellectual functioning. Our findings generally contrast findings from other studies conducted in sub-Saharan Africa. A Nigerian study by Sunmonu et al. [32], assessing intellectual performance of antiretroviral-naïve HIV-infected participants compared to a control group, found strong significant differences ($p<0.01$) with medium to large effect sizes (0.7 to 1.8). On the other hand, a study conducted in Uganda by Robertson et al [31] reported significant differences between HIV-infected subjects and controls for the backward digit span test ($p<0.001$) and tests of executive functioning (<0.01).

There are two potential explanations for these differences in findings. First, we recruited study participants (both the HIV-infected and controls) with ≤8 years of schooling unlike in the Nigerian and Ugandan studies [31, 32] where participants had up to 13 years of schooling. Using this criterion, we selected a group that was potentially at an elevated risk of poor performance on neurocognitive tests [48]. It is not surprising to see that the mean scores of our study participants compared to participants in the Ugandan study [31] for instance, were generally lower on the backward digit span test i.e. 2.2 versus 3.0 (for the HIV-infected groups) and 2.9 versus 3.5 (for the control groups). Even though our sample was of lower educational background, HIV-infected adults were significantly less literate than community controls. Accounting for the between-group educational differences among other factors, the initially observed unadjusted strong differences between adults living with HIV and community controls in non-verbal IQ and working memory tests disappeared.

Second, it may be plausible that our adult participants living with HIV were stable on antiretroviral therapy (ART). Only 4 participants out of the 78 with information on ART duration had used it for less than a year. ART is thought to delay the onset of milder neurocognitive impairment [49] (common even in the era of HAART) [10] or to improve the performance of patients with an existing HIV-associated neurocognitive dysfunction [50].

For peer review only - http://bmjopen.bmj.com/site/about/guidelines.xhtml

Either way, being stable on ART may have resulted in comparable neurocognitive test performance between adults living with HIV and community controls in our sample and ART-naivety in the study by Sunmonu et al. [32] may have explained why the authors observed strong significant group differences.

4.2 Mental health

In this study, living with HIV was significantly associated with increased depressive scores even after adjusting for covariates such as age, sex, education and socioeconomic status between the HIV-infected and control groups. Our finding corroborates findings reported from studies conducted in South Africa [51] and Nigeria [52]. Less education – a proxy for low socioeconomic status – has been found to be a risk factor for common mental disorders in the general population [53, 54]. In this study, we found meaningful adjusted differences in depressive scores comparing a sample of HIV-infected adults and community controls all with an already limited educational background. Our finding therefore suggests that a tendency towards scores indicative of depressive symptomatology may be because of HIV infection than a socioeconomic influence.

4.3 Neurocognitive, mental health and quality of life among adults living with HIV

Among adults living with HIV, we found no correlation between any of the neurocognitive scores and quality of life scores. However, depressive scores strongly correlated with quality of life scores and further exploration using multivariable linear regression analyses showed that high depressive scores were associated with poorer quality of life. This kind of association has previously been reported from studies conducted in other low-to-middle income settings [22, 55]. This finding suggests that interventions aimed at improving mental health are likely to contribute to enhanced quality of life among adults living with HIV in our setting.

Study limitations

This study has some limitations which should be considered when interpreting the findings. Firstly, as a cross-sectional study design, we cannot ascertain any causal relationship for the observed significant associations. Secondly, recruiting community controls based on their self-report of HIV seronegative status may have introduced some bias. Thirdly, because the larger study was primarily focussed on psychometric evaluation of the Neuroscreen, clinical

For peer review only - http://bmjopen.bmj.com/site/about/guidelines.xhtml

information of biomarkers i.e. viral load and CD4 cell count for adults living with HIV was not available for the current study and consequently not accounted for in our analyses. Relatedly, HIV disease staging, for which we have considered in our analysis, was collected historically (last known disease stage) and may not be reflective of a participant's disease state at the time of this study.

Conclusion

Our findings suggest that adults of low literacy levels living with HIV and on antiretroviral medication at the Kenyan Coast did not have cognitive deficits when compared to their uninfected counterparts. However, the mental health of HIV infected adults remains poorer and their quality of life may deteriorate when HIV and depressive symptoms co-occur. Interventions to enhance early identification and management of depression may promote better quality of life of, especially the socioeconomically disadvantaged patients living with HIV in our setting and other similar settings.

Contributor ship statement

MKN contributed to study design, did the formal analysis of the reported results, interpreted the results, and wrote the first draft of the manuscript. **PNM** supervised the field team during data collection, was in charge of quality control, and critically reviewed the manuscript. **PM** managed the study data and critically reviewed the manuscript. **MK** contributed to data collection and critically reviewed the manuscript. **CN** contributed to study design, interpretation of the results, and critically reviewed the manuscript. **AA** conceptualised the study, acquired funding for the study, contributed to the study design, interpretation of the results, and critically reviewed the manuscript. All the authors read and approved the final submitted version of the manuscript.

Competing interests

There are no competing interests for any author

Funding

This work was supported by funding from the Medical Research Council (Grant number MR/M025454/1) to AA. This award is jointly funded by the UK Medical Research Council (MRC) and the UK Department for International Development (DFID) under MRC/DFID concordant agreement and is also part of the EDCTP2 program supported by the European

For peer review only

Union. During this work, MKN was supported by the Wellcome Trust Master's Fellowship in Public Health and Tropical Medicine (Grant number 201310/Z/16/Z). The funders did not have a role in the design and conduct of the original study.

Data sharing statement

No additional data available. Anyone interested in accessing the data reported in this article, is free to write to the Data Governance Committee of the KEMRI Wellcome Trust Research Programme who will review the application and advise as appropriate, and ensure that uses are compatible with the consent obtained from participants for data collection. Requests can be sent to the coordinator of the Data Governance Committee using the following email: dgc@kemri-wellcome.org.

Acknowledgments

We wish to thank all our study participants for taking time, voluntarily, to participate in this study. We would also like to thank Katana Ngombo, Richard Karisa, Beatrice Kabunda, Judith Tumaini Dzombo and Khamisi Katana for their role in data collection. The authors would also like to thank the Director of Kenya Medical Research Institute (KEMRI) for the permission to publish this work.

References

1. Panos, G., et al., *Mortality and morbidity of HIV infected patients receiving HAART: a cohort study.* Current HIV Research, 2008. **6**(3): p. 257-260.
2. Johnson, L.F., et al., *Life expectancies of South African adults starting antiretroviral treatment: collaborative analysis of cohort studies.* PLoS medicine, 2013. **10**(4): p. e1001418.
3. Nsanzimana, S., et al., *Life expectancy among HIV-positive patients in Rwanda: a retrospective observational cohort study.* The Lancet Global Health, 2015. **3**(3): p. e169-e177.
4. Samji, H., et al., *Closing the gap: increases in life expectancy among treated HIV-positive individuals in the United States and Canada.* PloS one, 2013. **8**(12): p. e81355.
5. Nakagawa, F., M. May, and A. Phillips, *Life expectancy living with HIV: recent estimates and future implications.* Curr Opin Infect Dis, 2013. **26**(1): p. 17-25.
6. Watkins, C.C. and G.J. Treisman, *Cognitive impairment in patients with AIDS – prevalence and severity.* HIV/AIDS (Auckland, N.Z.), 2015. **7**: p. 35-47.

For peer review only - http://bmjopen.bmj.com/site/about/guidelines.xhtml

For peer review only

7. NIMH. *HIV/AIDS and Mental Health*. National Institute of Mental Health (NIMH), 2016. [23rd Jan 2018]; Available from: https://www.nimh.nih.gov/health/topics/hiv-aids/index.shtml.

8. McArthur, J.C., et al., *Human immunodeficiency virus-associated dementia: an evolving disease.* Journal of neurovirology, 2003. **9**(2): p. 205-221.

9. Saylor, D., et al., *HIV-associated neurocognitive disorder--pathogenesis and prospects for treatment.* Nat Rev Neurol, 2016. **12**(4): p. 234-48.

10. Heaton, R.K., et al., *HIV-associated neurocognitive disorders persist in the era of potent antiretroviral therapy: CHARTER Study.* Neurology, 2010. **75**(23): p. 2087-2096.

11. Ancuta, P., et al., *Microbial translocation is associated with increased monocyte activation and dementia in AIDS patients.* PloS one, 2008. **3**(6): p. e2516.

12. Habib, A.G., et al., *Neurocognitive impairment in HIV-1-infected adults in Sub-Saharan Africa: a systematic review and meta-analysis.* International Journal of Infectious Diseases, 2013. **17**(10): p. e820-e831.

13. Grant, I., *Neurocognitive disturbances in HIV.* International review of psychiatry, 2008. **20**(1): p. 33-47.

14. Heaton, R.K., et al., *HIV-associated neurocognitive disorders before and during the era of combination antiretroviral therapy: differences in rates, nature, and predictors.* Journal of neurovirology, 2011. **17**(1): p. 3-16.

15. Ogunrin, O. and F. Odiase, *Motor speed and reaction time in HIV/AIDS patients: a case-control study.* African journal of AIDS research, 2006. **5**(3): p. 217-220.

16. Simioni, S., et al., *Cognitive dysfunction in HIV patients despite long-standing suppression of viremia.* Aids, 2010. **24**(9): p. 1243-50.

17. Vivithanaporn, P., et al., *Neurologic disease burden in treated HIV/AIDS predicts survival A population-based study.* Neurology, 2010. **75**(13): p. 1150-1158.

18. Hinkin, C., et al., *Medication adherence among HIV+ adults effects of cognitive dysfunction and regimen complexity.* Neurology, 2002. **59**(12): p. 1944-1950.

19. Tozzi, V., et al., *Neurocognitive impairment influences quality of life in HIV-infected patients receiving HAART.* International journal of STD & AIDS, 2004. **15**(4): p. 254-259.

20. Heaton, R.K., et al., *The impact of HIV-associated neuropsychological impairment on everyday functioning.* Journal of the international neuropsychological society, 2004. **10**(3): p. 317-331.

For peer review only - http://bmjopen.bmj.com/site/about/guidelines.xhtml

21. Brandt, R., *The mental health of people living with HIV/AIDS in Africa: a systematic review.* African Journal of AIDS Research, 2009. **8**(2): p. 123-133.

22. Reis, R.K., et al., *Symptoms of depression and quality of life of people living with HIV/AIDS.* Revista latino-americana de enfermagem, 2011. **19**(4): p. 874-881.

23. Ciesla, J.A. and J.E. Roberts, *Meta-analysis of the relationship between HIV infection and risk for depressive disorders.* American Journal of Psychiatry, 2001. **158**(5): p. 725-730.

24. GIP. *Double stigma, double challenge: Mental health and HIV/AIDS in Central and Eastern Europe and the Newly Independent States. Global Inititative on Psychiatry (GIP)*, 2006. [15th January 2018]; Available from: http://www.bibalex.org/Search4Dev/files/417303/362887.pdf.

25. Singer, E.J. and A.D. Thames, *Neurobehavioral Manifestations of HIV/AIDS: Diagnosis and Treatment.* Neurologic clinics, 2016. **34**(1): p. 33-53.

26. Stutterheim, S.E., et al., *HIV-related stigma and psychological distress: the harmful effects of specific stigma manifestations in various social settings.* Aids, 2009. **23**(17): p. 2353-7.

27. Gonzalez, J.S., et al., *Depression and HIV/AIDS treatment nonadherence: a review and meta-analysis.* J Acquir Immune Defic Syndr, 2011. **58**(2): p. 181-7.

28. Chan, L.G., N. Kandiah, and A. Chua, *HIV-associated neurocognitive disorders (HAND) in a South Asian population-contextual application of the 2007 criteria.* BMJ open, 2012. **2**(1): p. e000662.

29. Yakasai, A.M., et al., *Prevalence and correlates of HIV-associated neurocognitive disorders (HAND) in Northwestern Nigeria.* Neurology research international, 2015. **2015**.

30. NACC. *Kenya HIV County Profiles - 2016*. National AIDS Control Council (NACC), 2016. [20 July 2017]; 49-52]. Available from: http://nacc.or.ke/wp-content/uploads/2016/12/Kenya-HIV-County-Profiles-2016.pdf.

31. Robertson, K.R., et al., *Pattern of neuropsychological performance among HIV positive patients in Uganda.* BMC Neurology, 2007. **7**(1): p. 8.

32. Sunmonu, T.A., et al., *Intellectual impairment in patients with newly diagnosed HIV infection in southwestern Nigeria.* BioMed research international, 2015. **2015**.

33. Scott, J.A.G., et al., *Profile: the Kilifi health and demographic surveillance system (KHDSS).* International journal of epidemiology, 2012. **41**(3): p. 650-657.

For peer review only - http://bmjopen.bmj.com/site/about/guidelines.xhtml

34. Von Elm, E., et al., *The Strengthening the Reporting of Observational Studies in Epidemiology (STROBE) statement: guidelines for reporting observational studies.* PLoS medicine, 2007. **4**(10): p. e296.

35. Abubakar, A., et al., *Socioeconomic status, anthropometric status, and psychomotor development of Kenyan children from resource-limited settings: a path-analytic study.* Early human development, 2008. **84**(9): p. 613-621.

36. Abubakar, A., et al., *Cumulative Psychosocial Risk is a Salient Predictor of Depressive Symptoms among Vertically HIV-Infected and HIV-Affected Adolescents at the Kenyan Coast.* Annals of global health, 2017.

37. Bech, P., et al., *The sensitivity and specificity of the Major Depression Inventory, using the Present State Examination as the index of diagnostic validity.* Journal of affective disorders, 2001. **66**(2): p. 159-164.

38. Olsen, L., et al., *The internal and external validity of the Major Depression Inventory in measuring severity of depressive states.* Psychological medicine, 2003. **33**(2): p. 351-356.

39. Otiende, M., et al., *Psychometric evaluation of the Major Depression Inventory among young people living in Coastal Kenya.* Wellcome Open Research, 2017. **2**.

40. Raven, J.C., J. Court, and J. Raven, *Manual for Raven's Progressive Matrices and Vocabulary Scales. The Coloured Progressive Matrices. Oxford Psychologist Press: London.* 1977.

41. Taylor, H.G., et al., *Postirradiation treatment outcomes for children with acute lymphocytic leukemia: Clarification of risks.* Journal of pediatric psychology, 1987. **12**(3): p. 395-411.

42. Wechsler, D., *Wechsler Adult Intelligence Scale–Revised manual. The Psychological Corporation: New York, NY.* 1981.

43. Riddle, T. and J. Suhr, *Extension of the contingency naming test to adult assessment: Psychometric analysis in a college student sample.* The Clinical Neuropsychologist, 2012. **26**(4): p. 609-625.

44. Hays, R.D. and L.S. Morales, *The RAND-36 measure of health-related quality of life.* Annals of medicine, 2001. **33**(5): p. 350-357.

45. Frempong-Ainguah, F. and A. Hill, *Reliability, validity and responsiveness of the short form-36 health survey: Findings from the women's health study of Accra, Ghana.* Revue quetelet/quetelet journal, 2017. **2**(2).

For peer review only

For peer review only - http://bmjopen.bmj.com/site/about/guidelines.xhtml

46. StataCorp LP, *Stata Statistical Software: Release 14. College Station, TX: StataCorp LP.* 2015.

47. Johnson, T.R., *Violation of the homogeneity of regression slopes assumption in ANCOVA for two-group pre-post designs: Tutorial on a modified Johnson-Neyman procedure.* Tutorials in quantitative methods for psychology 2016. **12**(3): p. 253-263.

48. Ngandu, T., et al., *Education and dementia: what lies behind the association?* Neurology, 2007. **69**(14): p. 1442-50.

49. Deutsch, R., et al., *AIDS-associated mild neurocognitive impairment is delayed in the era of highly active antiretroviral therapy.* Aids, 2001. **15**(14): p. 1898-1899.

50. Sacktor, N., et al., *Combination antiretroviral therapy improves psychomotor speed performance in HIV-seropositive homosexual men.* Neurology, 1999. **52**(8): p. 1640-1640.

51. Peltzer, K., et al., *Depression and Social Functioning among HIV-infected and Uninfected Persons in South Africa.* AIDS care, 2015. **27**(1): p. 41-46.

52. Chikezie, U., et al., *Depression among people living with human immunodeficiency virus infection/acquired immunodeficiency syndrome in Benin City, Nigeria: A comparative study.* Nigerian journal of clinical practice, 2013. **16**(2): p. 238-242.

53. Araya, R., et al., *Education and income: which is more important for mental health?* Journal of Epidemiology & Community Health, 2003. **57**(7): p. 501-505.

54. Yimam, K., Y. Kebede, and T. Azale, *Prevalence of common mental disorders and associated factors among adults in Kombolcha Town, Northeast Ethiopia.* Journal of Depression and Anxiety 2014. **1**: p. 2167-1044.

55. Charles, B., et al., *Association between stigma, depression and quality of life of people living with HIV/AIDS (PLHA) in South India – a community based cross sectional study.* BMC Public Health, 2012. **12**: p. 463-463.

For peer review only

For peer review only - http://bmjopen.bmj.com/site/about/guidelines.xhtml

STROBE 2007 (v4) Statement—Checklist of items that should be included in reports of *cross-sectional studies*

Section/Topic	Item #	Recommendation	Reported on page #
**Title and abstract**	1	(*a*) Indicate the study's design with a commonly used term in the title or the abstract	1
		(*b*) Provide in the abstract an informative and balanced summary of what was done and what was found	2
**Introduction**			
Background/rationale	2	Explain the scientific background and rationale for the investigation being reported	4-5
Objectives	3	State specific objectives, including any prespecified hypotheses	5
**Methods**			
Study design	4	Present key elements of study design early in the paper	5
Setting	5	Describe the setting, locations, and relevant dates, including periods of recruitment, exposure, follow-up, and data collection	5
Participants	6	(*a*) Give the eligibility criteria, and the sources and methods of selection of participants	6
Variables	7	Clearly define all outcomes, exposures, predictors, potential confounders, and effect modifiers. Give diagnostic criteria, if applicable	13 (Table 3)
Data sources/ measurement	8*	For each variable of interest, give sources of data and details of methods of assessment (measurement). Describe comparability of assessment methods if there is more than one group	8-9
Bias	9	Describe any efforts to address potential sources of bias	8
Study size	10	Explain how the study size was arrived at	6
Quantitative variables	11	Explain how quantitative variables were handled in the analyses. If applicable, describe which groupings were chosen and why	9/10
Statistical methods	12	(*a*) Describe all statistical methods, including those used to control for confounding	9/10
		(*b*) Describe any methods used to examine subgroups and interactions	N/A
		(*c*) Explain how missing data were addressed	Pairwise deletion using statistical methods (page 9)
		(*d*) If applicable, describe analytical methods taking account of sampling strategy	9

For peer review only

		(*e*) Describe any sensitivity analyses	N/A
**Results**			
Participants	13*	(a) Report numbers of individuals at each stage of study—eg numbers potentially eligible, examined for eligibility, confirmed eligible, included in the study, completing follow-up, and analysed	7
		(b) Give reasons for non-participation at each stage	7
		(c) Consider use of a flow diagram	7
Descriptive data	14*	(a) Give characteristics of study participants (eg demographic, clinical, social) and information on exposures and potential confounders	10 - 11 (Table 1)
		(b) Indicate number of participants with missing data for each variable of interest	10 - 11 (Table 1)
Outcome data	15*	Report numbers of outcome events or summary measures	11-12
Main results	16	(*a*) Give unadjusted estimates and, if applicable, confounder-adjusted estimates and their precision (eg, 95% confidence interval). Make clear which confounders were adjusted for and why they were included	12 -13
		(*b*) Report category boundaries when continuous variables were categorized	N/A
		(*c*) If relevant, consider translating estimates of relative risk into absolute risk for a meaningful time period	N/A
Other analyses	17	Report other analyses done—eg analyses of subgroups and interactions, and sensitivity analyses	N/A
**Discussion**			
Key results	18	Summarise key results with reference to study objectives	11-13
Limitations	19	Discuss limitations of the study, taking into account sources of potential bias or imprecision. Discuss both direction and magnitude of any potential bias	15
Interpretation	20	Give a cautious overall interpretation of results considering objectives, limitations, multiplicity of analyses, results from similar studies, and other relevant evidence	16
Generalisability	21	Discuss the generalisability (external validity) of the study results	16
**Other information**			
Funding	22	Give the source of funding and the role of the funders for the present study and, if applicable, for the original study on which the present article is based	16

*Give information separately for cases and controls in case-control studies and, if applicable, for exposed and unexposed groups in cohort and cross-sectional studies.

Note: An Explanation and Elaboration article discusses each checklist item and gives methodological background and published examples of transparent reporting. The STROBE checklist is best used in conjunction with this article (freely available on the Web sites of PLoS Medicine at http://www.plosmedicine.org/, Annals of Internal Medicine at http://www.annals.org/, and Epidemiology at http://www.epidem.com/). Information on the STROBE Initiative is available at www.strobe-statement.org.

For peer review only

BMJ Open

Neurocognitive and mental health outcomes and association with quality of life among adults living with HIV: A cross-sectional focus on a low-literacy population from Coastal Kenya

Journal:	*BMJ Open*
Manuscript ID	bmjopen-2018-023914.R1
Article Type:	Research
Date Submitted by the Author:	18-Jul-2018
Complete List of Authors:	Nyongesa, Moses; Centre for Geographic Medicine Research Coast, NeuroAssessment<br>Mwangala, Patrick ; Centre for Geographic Medicine Research Coast<br>Mwangi, Paul; Centre for Geographic Medicine Research Coast<br>Kombe, Martha; Centre for Geographic Medicine Research Coast<br>Newton, Charles; KEMRI-Wellcome Trust Research Programme, Centre for Geographic Medicine Research -Coast, Clinical Research<br>Abubakar, Amina A.; Centre for Geographic Medicine Research Coast
**Primary Subject Heading**:	HIV/AIDS
Secondary Subject Heading:	Mental health, Global health, Neurology
Keywords:	HIV & AIDS < INFECTIOUS DISEASES, Neurocognitive performance, MENTAL HEALTH, QUALITY OF LIFE, LITERACY, ADULTS

SCHOLARONE™ Manuscripts

Neurocognitive and mental health outcomes and association with quality of life among adults living with HIV: A cross-sectional focus on a low-literacy population from Coastal Kenya

Moses K. Nyongesa[1*], Patrick N. Mwangala[1], Paul Mwangi[1], Martha Kombe[1], Charles R. Newton[1, 2, 3], Amina Abubakar[1, 2, 3]

1 Neuroassessment Group, KEMRI-Wellcome Trust Research Programme, Center for Geographic Medicine Research (Coast), Kilifi, Kenya

2 Department of Psychiatry, University of Oxford, Oxford, United Kingdom

3 Department of Public Health, Pwani University, Kilifi, Kenya

 Corresponding author. E-mail address: Mkachama@kemri-wellcome.org, KEMRI-Wellcome Trust Research Programme, Center for Geographic Medicine Research (Coast), PO Box 230 (80108) Kilifi, Kenya.

For peer review only

Target Journal: BMJ Open

Word Count: 3741

Abstract

Objectives: Our aim was to compare the neurocognitive performance and mental health outcome of adults living with HIV on antiretroviral therapy with that of community controls; all of low-literacy. Furthermore, we also wanted to explore the relationship of these outcomes with quality of life among adults living with HIV.

Study design: This was a descriptive cross-sectional study.

Setting: The study was conducted in Kilifi County, a region located at the Kenyan Coast.

Participants: The participants consisted of a consecutive sample of 84 adults living with HIV and 83 randomly selected community controls all with ≤8 years of schooling. All participants were assessed for non-verbal intelligence, verbal working memory and executive functioning. The Major Depression Inventory (MDI) and a quality of life measure (RAND SF-36) were also administered.

Results: Using analysis of covariance (ANCOVA), we found no statistically significant group differences between adults living with HIV and community controls in all the neurocognitive tests except for a marginal difference in the non-verbal intelligence test ($F\ (1, 158) = 3.83,\ p=0.05$). However, depressive scores of adults living with HIV were significantly higher than those of controls ($F\ (1, 158) = 11.56,\ p<0.01$). Also, quality of life scores of adults living with HIV were significantly lower than those of controls ($F\ (1, 158) = 4.62,\ p=0.03$). For the HIV-infected group, results from multivariable linear regression analysis showed that increasing depressive scores were significantly associated with poorer quality of life ($\beta=-1.17$, *95% CI -1.55, -0.80*; $p<0.01$).

Conclusion: Our findings suggest that adults of low literacy levels living with HIV and on antiretroviral medication at the Kenyan Coast do not have significant cognitive deficits compared to their uninfected counterparts. However, their mental health, compared to that of HIV uninfected adults, remains poorer and their quality of life may deteriorate when HIV and depressive symptoms co-occur.

Keywords: HIV/AIDs, neurocognitive performance, mental health, quality of life, literacy, adults, Kenya

For peer review only - http://bmjopen.bmj.com/site/about/guidelines.xhtml

Strengths and limitations of this study

- This is one of the few studies simultaneously exploring cognitive, mental health and quality of life outcomes among HIV-infected sample from sub Saharan Africa.
- We also focus specifically on a low-literacy sample
- The key limitation is the cross-sectional nature of our study design, therefore, we cannot ascertain any causal relationship

For peer review only

For peer review only - http://bmjopen.bmj.com/site/about/guidelines.xhtml

1.0 Introduction

The introduction of highly active antiretroviral therapy (HAART) – more than three decades now – has resulted to significant declines in HIV/AIDs related morbidity and mortality [1]. An increase in life expectancy among individuals living with HIV and on HAART has also been observed in both low [2, 3] and high-resourced [4] settings. For HIV patients who are optimally treated with antiretroviral medication, their life expectancy may approach that of HIV uninfected population [5]. Despite this achievement in longevity, neurocognitive disturbance and psychological problems remain as important health issues of concern among people living with HIV/AIDs (PLWH) [6, 7].

Neurocognitive impairment (NCI) is a common complication following HIV infection [8, 9], even with the advent of HAART [10]. The NCI associated with HIV, also known as HIV-associated neurocognitive disorder (HAND), arises when HIV infected monocytes and macrophages infiltrate the brain by crossing the blood-brain barrier, to infect and activate microglia causing inflammation of the central nervous system and progressive cognitive dysfunction (from involvement of the cortical and subcortical regions) [9, 11]. In sub-Saharan Africa (SSA), where there is a substantial burden of HIV infection compared to other regions of the world, prevalence estimates for HAND as high as 42% (pre-HAART) and 30% (post-HAART) have been reported [12]. Some of the cognitive deficits described among PLWH, through neuropsychological testing, include impairment in attention/concentration, processing speed and executive functioning, as well as problems with motor functioning, learning and memory [13-15]. For PLWH on HAART and with well-controlled viremia, mild NCI is much more common than severe cognitive impairment i.e. HIV-associated dementia [10, 16]. There are significant clinical and functional consequences of having even mild NCI including: increased risk of mortality [17]; poor medication adherence [18]; decreased quality of life [19]; and disruptions to everyday functioning [20].

Mental disorders are also common among PLWH, depression being the most prevalent [21, 22]. A review of the mental health of adults living with HIV in Africa [21] reported estimates for depressive symptoms of over 30% and as high as 64% from different reviewed studies. Furthermore, major depressive disorder has been found to occur almost twice as frequent among PLWH compared to the HIV-uninfected subjects [23].

For peer review only - http://bmjopen.bmj.com/site/about/guidelines.xhtml

There are many reasons as to why PLWH may experience mental disorders frequently, particularly depressive symptoms. Firstly, is the difficulty of living with a chronic, life-threatening illness. This adjustment difficulty – presenting as psychological distress – may occur after receiving HIV positive diagnosis; when HIV symptoms begin to develop; at the start of lifelong antiretroviral medication; and also in terminal care [24]. Secondly, the direct effects of HIV virus on the central nervous system [25], may lead to behavioural changes resulting into symptoms of depression. Thirdly, such psychological disturbance may also be because of HIV-related stigma [26]. PLWH with comorbid mental illnesses may present with poor quality of life [22] and non-adherence to antiretroviral medication [27]. Non-adherence may lead to faster disease progression or increased HIV/AIDs related mortality.

Cognitive and mental health outcomes of PLWH and their relationship with quality of life have gained increasing attention recently, with most work being conducted and reported from highly resourced countries, but limited (although increasing) reports from SSA, where the burden of HIV/AIDS is substantial. To add to the body of research evidence from SSA, we evaluated the neurocognitive test performance and mental health of adults living with HIV on antiretroviral medication compared to community controls, specifically focusing on a low-literacy population at the Kenyan Coast. Less education among PLWH has been associated with an increased risk for symptomatic HAND [28, 29]. We also set out to explore the relationship between each of these outcomes with quality of life among adults living with HIV.

2.0 Methods

2.1 Study site:

This study was undertaken at the Centre for Geographic Medicine Research-Coast (CGMR-C), Kilifi County, Kenya between November 2016 and March 2017. The Centre has a Neuro-assessment unit equipped for assessing a wide range of neurocognitive and neuropsychiatric outcomes in children, adolescents and adults living with chronic illnesses such as HIV and epilepsy. Interviews and tests are carried out by trained and experienced research assistants. In Kilifi County, the estimated overall adult HIV prevalence is 4.5% with prevalence being higher among females (6.4%) than males (2.7%) [30].

2.2 Study design:

For peer review only - http://bmjopen.bmj.com/site/about/guidelines.xhtml

This was a cross-sectional study aimed at investigating neurocognitive functioning, mental health and their association with quality of life in an adult sample. This study was embedded in a larger study whose aim was to validate a tablet-based android application (App) – the NeuroScreen, Swahili version – screening for neurocognitive impairment among chronically ill adults in Kilifi. In the NeuroScreen study, only participants with ≤8 years of schooling (up to primary level of education in the Kenyan education system) were included with the assumption that if the App works with the less literate individuals in Kilifi, then it should work well with those who are better educated.

2.3 Sample size estimation:

In this current study, the sample size (n=167) included 84 participants living with HIV and 83 community controls. This sample size was calculated to detect differences in cognitive outcomes between adults living with HIV and controls with 90% power using previously reported effect sizes [31, 32].

2.4 Recruitment and criteria for inclusion:

Participants living with HIV: Adults living with HIV were recruited from the Comprehensive Care and Research Clinic (CCRC), an HIV specialised clinic at the Kilifi County Hospital. Selection and booking for assessment date was done through consecutive sampling of clients on their arrival at the clinic. Participants were eligible for inclusion in the study if they: i) were 20 to 50 years old; ii) had a confirmed diagnosis of HIV; iii) had no more than primary level of education (an inclusion criterion for the larger study validating the NeuroScreen); iv) could understand and communicate in Kiswahili (the official national language of Kenya) with ease; v) provided informed consent; and vi) had no obvious symptoms of acute illness on the day of assessment.

Community Controls: community controls were identified through the Kilifi Health and Demographic Surveillance System (KHDSS) by random selection and later approached at home and requested to take part in the study. Details about the KHDSS can be found elsewhere [33]. Those who consented to taking part in the study were referred to CGMR-C for assessment if they met the eligibility criteria as above, except that of having an HIV diagnosis. These participants had to self-report a seronegative test result in the last year. We did not test for HIV among these controls, and we acknowledge that in randomly selecting them, about 3 or 4 out of the 84 may have been HIV-infected considering the adult HIV prevalence in Kilifi County, Kenya [30]. However, through a detailed clinical examination

For peer review only - http://bmjopen.bmj.com/site/about/guidelines.xhtml

(see study procedures below), we only included participants having no symptoms of an acute illness. Using this criterion, we expected very few cases of asymptomatic HIV-infection among our controls that is unlikely to invalidate our findings.

Figure 1 is a flowchart showing the recruitment process for both adults living with HIV and community controls in line with the STROBE guidelines for reporting observational studies [34].

Figure 1 Flowchart of participant recruitment

2.5 Study Procedures:

Every consented participant underwent a detailed medical history and symptomatology clinical examination. For controls, other than ruling out symptoms of an obvious acute illness for participation, symptomatologic evaluation was also done to rule out the possibilities of HIV infection. A set of questionnaires were then administered to capture information about sociodemographic and economic background, mental health and quality of life. Cognitive assessments were paper and pencil and computer tablet-based. Data collection was conducted by a trained team of research assistants.

2.6 Measures:

The measures listed below were all administered to both adults living with HIV and community controls. For both groups, all measures were administered in the same order, by the same assessor (who was blinded) and under the same administration environment.

Social demographic and asset index forms: These were used to collect information on participant's age, sex, marital status, level of education, religion and socioeconomic status. The asset index has been reliably used to assess socioeconomic status over time in studies conducted in this setting [35, 36]. The asset index items ask for ownership of disposable assets by participants (or their family) such as radio, television, bicycle or motorbike et cetera.

Major Depression Inventory (MDI): This is a self-report measure of depressive symptoms that examines moods experienced by an individual during the past 2 weeks. It consists of 12 items, although only 10 items are scored since for items 8 (a and b) and 10 (a and b) only an alternative with the highest score is considered. Items are scored on a 6-point Likert scale from 0 (at no time) to 5 (all the time) with the total score ranging from 0 to 50 [37]. The MDI can be used both as a diagnostic instrument for major depression according to the Diagnostic

For peer review only - http://bmjopen.bmj.com/site/about/guidelines.xhtml

For peer review only

and Statistical Manual for Mental Disorders, version four (DSM-IV) and as a depression rating scale. As a rating scale, MDI total score of 20-24 is indicative of mild depressive symptoms, a score of 25-29 is indicative of moderate depressive symptoms whereas a score ≥ 30 indicates severe depressive symptoms [38]. This measure, adapted to Swahili, yielded reliable psychometric properties among young adults in Kilifi [39].

Measures of cognitive performance: In this study, three tests were administered to assess cognitive functioning among study participants i.e. the Raven's Standard Progressive Matrices (RSPM) [40], Contingency Naming Test (CNT) [41] and the Backward Digit span test [42]. RSPM is a non-verbal standardized test of intelligence quotient (IQ) comprising of five series with 12 items without limit in execution time. The CNT, initially developed to assess aspects of executive functioning such as processing speed and response inhibition in children, has shown good psychometric reliability when extended to the adult population [43]. In the CNT test, a participant is required to name the colour or shape of a series of stimuli, as fast and accurately as possible, according to different rules. The Backward Digit Span test assesses verbal working memory [42]. A list of random numbers is read aloud to the participant who is then required to recall the items in a reverse order.

The RAND 36-Item Health Survey 1.0 (RAND SF-36): This is the most widely used generic measure of health-related quality of life. It assesses 8 health concepts: physical functioning, role limitations due to physical health problems, role limitations due to emotional problems, social functioning, emotional wellbeing, energy/fatigue, pain, and general health perceptions [44]. Scoring the tool involves various steps. Firstly, pre-coded numeric values are converted to percentages using a predetermined scoring key. Secondly, items in the same scale are summed and then averaged to give the 8 different sub-scale scores. Lastly, to generate a total score, all item scores are summed and then averaged. Higher scores (as percentage) define a more favourable state of health. RAND SF-36 has been found to be reliable in SSA [45]. For this study, the English questionnaire was forward translated into Kiswahili by two independent translators. Back translation into English was then undertaken by two other independent back translators (unaware of the original version). The final version was obtained following a harmonization process by a team of experts and incorporation of changes resulting from a cognitive interview process and pretesting procedures. In the current study, Cronbach's alpha for this measure was excellent at 0.93.

2.7 Ethical consideration:

For peer review only - http://bmjopen.bmj.com/site/about/guidelines.xhtml

This study was approved by the Kenya Medical Research Institute Scientific and Ethics Research Unit (Ref no: KEMRI/SERU/CGMR-C/030/3187). Permission to work in the comprehensive care clinic was also obtained from the Ministry of Health, Kilifi County government. Voluntary and written informed consent was obtained from all study participants after a detailed explanation of the study had been made to them.

2.8 Patient and Public Involvement

Patients were not involved in the design and conduct of the study

2.9 Statistical analyses:

All analyses were carried out on STATA (version 14.0) statistical software package [46]. Frequencies, percentages, mean or median were used to describe sample characteristics. Chi-square tests (with Fisher's exact test for continuity correction where appropriate) and independent student's t-test (or Wilcoxon rank sum test where appropriate) were used to compare group differences between categorical and continuous variables respectively. Analysis of covariance (ANCOVA) was used to control for covariates of interest when looking at group differences in neurocognitive test performance, depressive and quality of life scores between adults living with HIV and community controls. We checked for ANCOVA's homogeneity of regression slope assumption using a method suggested by Johnson [47]. In an analysis involving only the HIV-infected group, we used correlational and multivariable linear regression analyses to explore potential associations between neurocognitive test performance, depressive scores and quality of life scores. All tests in our analyses were considered statistically significant at $p < 0.05$.

3.0 Results.

Sample characteristics: Table 1 below describes sample characteristics by HIV status (i.e. adults living with HIV versus controls). The mean age of the 167 study participants who took part in the study was *36.95* (standard deviation [SD]= 8.65). Majority of the study participants were: females *(71.3%)*; and married (*63.9%*). Less than a quarter of study participants (*16.4%)* did not have any formal education. As shown in Table 1, adults living with HIV were significantly different from controls in all sociodemographic characteristics except for socioeconomic status (*p=0.92*). Nutritional status of the study participants (as indicated by body mass index and mid upper-arm circumference) and the clinical information

For peer review only - http://bmjopen.bmj.com/site/about/guidelines.xhtml

of adults living with HIV are also presented in Table 1. We also present observations with missing values.

Table 1: **Sample characteristics of adult participants living with HIV and community controls (n=167) along with clinical characteristics of participants living with HIV.**

Sample Characteristics	Total sample	HIV infected n=84	Controls n=83	P value
	n (%) or mean(SD) or Median (IQR)			
**Age (in years; mean [SD])**	37.0 (8.7)	40.1 (6.4)	33.8 (9.5)	**<0.01$^{\Psi}$**
**Sex**				
Female	119 (71.3)	66 (78.6)	53 (63.9)	**0.04**
Male	48 (28.7)	18 (21.4)	30 (36.1)	
**Marital status OM=1**				
Never married	27 (16.3)	8 (9.5)	19 (23.2)	**<0.01***
Married	106 (63.9)	47 (56.0)	59 (72.0)	
Separated/Divorced/Widowed	33 (19.9)	29 (34.5)	4 (4.9)	
**Education OM=2**				
None	27 (16.4)	20 (24.1)	7 (8.5)	**<0.01**
Primary incomplete	73 (44.2)	47 (56.6)	26 (31.7)	
Primary complete	65 (39.4)	16 (19.3)	49 (59.8)	
**SES Asset Index (median [IQR])**	2.0 (1.0, 3.0)	1.0 (1.0, 3.0)	2.0 (1.0, 2.0)	0.92$^{\Omega}$
**BMI (median [IQR])**	22.4 (20.2, 26.2)	22.4 (19.0, 26.7)	22.3 (20.5, 25.9)	0.67$^{\Omega}$
**MUAC (median [IQR])**	26.9 (25.0, 29.3)	26.7 (24.2, 29.0)	27.1 (25.2, 29.4)	0.33$^{\Omega}$
**Severity of depressive symptoms**				
No depressive symptom	147 (88.0)	69 (82.1)	78 (94.0)	0.06*
Mild depressive symptoms	9 (5.4)	8 (9.5)	1 (1.2)	
Moderate depressive symptoms	6 (3.6)	4 (4.8)	2 (2.4)	
Severe depressive symptoms	5 (3.0)	3 (3.6)	2 (2.4)	
**HIV-related clinical characteristics**				
**HIV disease staging OM=6**				
Stage 1	NA	38 (48.7)	NA	NA
Stage 2	NA	27 (34.6)	NA	NA
Stage 3	NA	13 (16.7)	NA	NA
**Number of ARV medication OM=7**				
Two	NA	56 (72.7)	NA	NA
Three	NA	21 (27.3)	NA	NA
**Duration on ARV**				
< 1 year	NA	4 (5.1)	NA	NA
1-5 years	NA	29 (37.2)	NA	NA
6-10 years	NA	36 (46.2)	NA	NA
> 10 years	NA	9 (11.5)	NA	NA

N/B: Percentages are exclusive of missing values. For observations with missing values, the number of missing values is indicated in the table as **OM** (observations with missing values). P-values are based on chi-squared statistic except for: ***** - chi-square based on Fisher's exact test; $^{\Omega}$ - based on Mann-Whitney U-test; **Ψ** – based on independent t-test; **SD** – standard deviation; **IQR** – inter quartile range; **SES** – socioeconomic status; **CNT** – Contingency Naming Test; **QoL** -Quality of life; **BMI** – Body Mass Index; **MUAC** – Mid upper-arm circumference **ARV** – antiretroviral; **NA** – Not Applicable

3.1 Neurocognitive performance, mental health and quality of life of adults living with HIV compared to community controls

For peer review only - http://bmjopen.bmj.com/site/about/guidelines.xhtml

Neurocognitive performance: in the crude analysis, adults living with HIV compared to controls had significantly lower mean non-verbal IQ scores as measured by the RSPM (*p<0.01; Table 2*) and verbal working memory scores i.e. lower mean total correct scores (*p=0.01; Table 2*) and mean score of highest set of digits reached (*p<0.01; Table 2*) of the backward digit span test. However, no significant difference was observed between adults living with HIV and controls in executive functioning as assessed by CNT total error scores (*p=0.32; Table 2*). Using ANCOVA adjusting for sex, age, level of education and socioeconomic index as covariates, there was no significant differences between adults living with HIV and community controls for all neurocognitive test scores except non-verbal IQ scores where a marginal difference was observed, $F\ (1,\ 158) = 3.83$, p=0.05, with a small effect size (*0.29; Table 2*).

Mental health: Twenty (12.0%) of study participants met the criteria for mild to severe depressive symptoms (15 being PLWH and 5 being community controls) as measured by the MDI. Adults living with HIV had significantly higher mean depressive scores than controls (*p<0.01; Table 2*) in the crude analysis. ANCOVA controlling for sex, age, education and socioeconomic status (*Table 2*) still found significant differences between adults living with HIV and community controls, $F\ (1,\ 158) = 11.56,\ P<0.01$.

Quality of life: in the crude analysis, adults living with HIV compared to controls had significantly lower mean quality of life scores as measured by the RAND SF-36 (*p<0.01; Table 2*). Controlling for sex, age, education and socioeconomic status in ANCOVA (*Table 2*), there was still a statistically significant difference between adults living with HIV and community controls, $F\ (1,\ 158) = 4.62,\ p=0.03$.

Table 2: Unadjusted and adjusted analyses for neurocognitive test performance, mental health and quality of life outcomes comparing adults living with HIV and community controls

	Unadjusted analysis			Adjusted analysis*					
**Outcome**	**HIV score**	**Control score**	**p - value**	**HIV score**	**Control score**	**F - statistic**	**p - value**	**eta-squared (%)**	**ES**
**Neurocognitive test performance**									
CNT total errors	2.82 (0.92)	2.68 (0.85)	0.32	2.89 (0.12)	2.79 (0.12)	0.38	0.54	6.5	0.09
Ravens total score	16.82 (5.39)	19.81 (7.28)	**<0.01**	16.72 (0.82)	18.92 (0.83)	3.83	**0.05**	14.6	0.29
Digit Span -total correct score	5.06 (2.81)	6.41 (3.32)	**0.01**	4.85 (0.39)	5.42 (0.41)	1.05	0.31	18.7	0.16
Digit Span - highest set of digits reached#	2.19 (1.26)	2.90 (1.44)	**<0.01**	2.06 (0.17)	2.47 (0.18)	3.05	0.08	21.9	0.27
**Mental health**									
Depressive scores	11.80 (8.38)	7.96 (7.32)	**<0.01**	12.23 (1.03)	7.46 (1.04)	11.56	**<0.01**	11.71	0.50

For peer review only - http://bmjopen.bmj.com/site/about/guidelines.xhtml

**Quality of life**									
QoL total score	70.38 (17.18)	78.07 (14.72)	**<0.01**	72.56 (2.12)	78.78 (2.14)	4.62	**0.03**	9.64	0.32

NB: All scores presented as mean (SD), unadjusted analysis; and mean (SE), adjusted analysis; **ES** – Cohen's d effect size
***ANCOVA** adjusted for sex, age, level of education and socioeconomic index
there were 8 sets with a series of random digits; sets 1, 2 and 3 had a length of 3 digits, sets 4 through 8 had digit lengths of 4 through 8 respectively

3.2 Correlation of neurocognitive, depressive and quality of life scores in the HIV-infected group

There was no statistically significant correlation between any of the neurocognitive scores and quality of life scores i.e. total correct score (*r=-0.05, p= 0.63*), score of the highest set of digits reached (*r=-0.01, p=0.96*), CNT's total error scores (*r=0.08, p=0.45*) or Ravens total score (*rho=-0.05, p=0.68*). However, correlation analysis revealed a significant correlation between depressive and quality of life scores (*rho=-0.67; p<0.01*). The reported results in the subsequent sections will therefore focus on findings from analyses that were carried out based on the observed significant correlation between depressive and quality of life scores.

3.3 Association between depressive symptoms and quality of life in the HIV-infected group

Exploring further the observed significant correlation between depressive and quality of life scores among adults living with HIV, a one-unit increase in depressive score was associated with 1.2 reduction in quality of life scores in the univariate linear regression analyses (*p<0.01; Table 3*). Adjusting for age, sex, number of and duration on antiretroviral medication and HIV disease staging, there was still strong evidence of an association between elevated depressive scores and poorer quality of life (*β=-1.17, 95% CI -1.55, -0.80; p<0.01; Table 3*).

For peer review only

	Univariate analysis			Multivariable analysis		
**Exposure variable**	**unadjusted β Coefficient (95% CI)**	**p-value**	**R-squared (%)**	**adjusted β Coefficient* (95% CI)**	**p-value**	**R-squared (%)**
Depressive scores	-1.17 (-1.53, -0.80)	**<0.01**	36.70	-1.17 (-1.55, -0.80)	**<0.01**	45.19
**Adjusted for:**						
Age				-0.20 (-0.77, 0.37)	0.48	
Sex (male vs female)				7.02 (-1.18, 15.22)	0.09	
Number of ARV medication (3 vs 2)				-4.49 (-12.32, 3.33)	0.26	
HIV staging						

For peer review only - http://bmjopen.bmj.com/site/about/guidelines.xhtml

Stage 2 vs 1	-2.63 (-10.04, 4.77)	0.48
Stage 3 vs 1	-8.60 (-18.12, 0.91)	0.08
Duration on ARV		
1-5 years vs <1 year	7.83 (-6.63, 22.29)	0.28
6-10 years vs <1 year	9.29 (-5.16, 23.73)	0.20
>10 years vs <1 year	11.28 (-4.87, 27.43)	0.17

NB: * Linear regression adjusted coefficient for quality of life; **ARV** – antiretroviral

Table 3: Univariate and multivariable linear regression analyses for the association between elevated depressive scores (exposure) and poorer quality of life (outcome) among adults living with HIV in Coastal Kenya, n=72

For peer review only

4.0 Discussion

In this study, we were interested in evaluating neurocognitive and mental health outcomes of adults living with HIV compared to community controls, specifically focusing on a low-literacy sample from the Kenyan Coast. We also wanted to explore the relationship between each of these outcomes and quality of life among the HIV-infected adult group.

4.1 Neurocognitive test performance

In summary, we did not observe main effect of HIV infection on most of the neurocognitive tests except that of non-verbal intelligence when comparing adults living with HIV and community controls all with lower educational background. For non-verbal intelligence, the adjusted group difference was marginally significant with a small effect size (0.3). We think that this marginal difference may be due to impaired premorbid intellectual functioning. Our findings generally contrast findings from other studies conducted in sub-Saharan Africa. A Nigerian study by Sunmonu et al. [32], assessing intellectual performance of antiretroviral-naïve HIV-infected participants compared to a control group, found strong significant differences ($p<0.01$) with medium to large effect sizes (0.7 to 1.8). On the other hand, a study conducted in Uganda by Robertson et al [31] reported significant differences between HIV-infected subjects and controls for the backward digit span test ($p<0.001$) and tests of executive functioning (<0.01).

There are two potential explanations for these differences in findings. First, we recruited study participants (both the HIV-infected and controls) with ≤8 years of schooling unlike in the Nigerian and Ugandan studies [31, 32] where participants had up to 13 years of schooling. Using this criterion, we selected a group that was potentially at an elevated risk of poor performance on neurocognitive tests [48]. It is not surprising to see that the mean scores

For peer review only - http://bmjopen.bmj.com/site/about/guidelines.xhtml

of our study participants compared to participants in the Ugandan study [31] for instance, were generally lower on the backward digit span test i.e. 2.2 versus 3.0 (for the HIV-infected groups) and 2.9 versus 3.5 (for the control groups). Even though our sample was of lower educational background, HIV-infected adults were significantly less literate than community controls. Accounting for the between-group educational differences among other factors, the initially observed unadjusted strong differences between adults living with HIV and community controls in non-verbal IQ and working memory tests disappeared.

Second, it may be plausible that our adult participants living with HIV were stable on antiretroviral therapy (ART). Only 4 participants out of the 78 with information on ART duration had used it for less than a year. ART is thought to delay the onset of milder neurocognitive impairment [49] (common even in the era of HAART) [10] or to improve the performance of patients with an existing HIV-associated neurocognitive dysfunction [50]. Either way, being stable on ART may have resulted in comparable neurocognitive test performance between adults living with HIV and community controls in our sample and ART-naivety in the study by Sunmonu et al. [32] may have explained why the authors observed strong significant group differences.

4.2 Mental health

In this study, living with HIV was significantly associated with increased depressive scores even after adjusting for covariates such as age, sex, education and socioeconomic status between the HIV-infected and control groups. Our finding corroborates findings reported from studies conducted in South Africa [51] and Nigeria [52]. Less education – a proxy for low socioeconomic status – has been found to be a risk factor for common mental disorders in the general population [53, 54]. In this study, we found meaningful adjusted differences in depressive scores comparing a sample of HIV-infected adults and community controls all with an already limited educational background. Our finding therefore suggests that a tendency towards scores indicative of depressive symptomatology may be because of HIV infection than a socioeconomic influence.

4.3 Neurocognitive, mental health and quality of life among adults living with HIV

Among adults living with HIV, we found no correlation between any of the neurocognitive scores and quality of life scores. However, depressive scores strongly correlated with quality of life scores and further exploration using multivariable linear regression analyses showed that high depressive scores were associated with poorer quality of life. This kind of

For peer review only - http://bmjopen.bmj.com/site/about/guidelines.xhtml

association has previously been reported from studies conducted in other low-to-middle income settings [22, 55]. This finding suggests that interventions aimed at improving mental health are likely to contribute to enhanced quality of life among adults living with HIV in our setting.

Study limitations

This study has some limitations which should be considered when interpreting the findings. Firstly, as a cross-sectional study design, we cannot ascertain any causal relationship for the observed significant associations. Secondly, recruiting community controls based on their self-report of HIV seronegative status may have introduced some bias. Thirdly, because the larger study was primarily focussed on psychometric evaluation of the Neuroscreen, clinical information of biomarkers i.e. viral load and CD4 cell count for adults living with HIV was not available for the current study and consequently not accounted for in our analyses. Relatedly, HIV disease staging, for which we have considered in our analysis, was collected historically (last known disease stage) and may not be reflective of a participant's disease state at the time of this study. Lastly, we recruited and administered neurocognitive tests to a low literacy sample. The potential lack of familiarity with neurocognitive test requirement/demands by our study participants, because of this biased selection, limits the generalizability of our finding. Our findings should be interpreted in the context of a low-literacy population.

Conclusion

Our findings suggest that adults of low literacy levels living with HIV and on antiretroviral medication at the Kenyan Coast did not have cognitive deficits when compared to their uninfected counterparts. However, the mental health of HIV infected adults remains poorer and their quality of life may deteriorate when HIV and depressive symptoms co-occur. Interventions to enhance early identification and management of depression may promote better quality of life of, especially the socioeconomically disadvantaged patients living with HIV in our setting and other similar settings.

Contributor ship statement

MKN contributed to study design, did the formal analysis of the reported results, interpreted the results, and wrote the first draft of the manuscript. **PNM** supervised the field team during

For peer review only - http://bmjopen.bmj.com/site/about/guidelines.xhtml

data collection, was in charge of quality control, and critically reviewed the manuscript. **PM** managed the study data and critically reviewed the manuscript. **MK** contributed to data collection and critically reviewed the manuscript. **CN** contributed to study design, interpretation of the results, and critically reviewed the manuscript. **AA** conceptualised the study, acquired funding for the study, contributed to the study design, interpretation of the results, and critically reviewed the manuscript. All the authors read and approved the final submitted version of the manuscript.

Competing interests

There are no competing interests for any author

Funding

This work was supported by funding from the Medical Research Council (Grant number MR/M025454/1) to AA. This award is jointly funded by the UK Medical Research Council (MRC) and the UK Department for International Development (DFID) under MRC/DFID concordant agreement and is also part of the EDCTP2 program supported by the European Union. During this work, MKN was supported by the Wellcome Trust Master's Fellowship in Public Health and Tropical Medicine (Grant number 201310/Z/16/Z). The funders did not have a role in the design and conduct of the original study.

Data sharing statement

No additional data available. Anyone interested in accessing the data reported in this article, is free to write to the Data Governance Committee of the KEMRI Wellcome Trust Research Programme who will review the application and advise as appropriate, and ensure that uses are compatible with the consent obtained from participants for data collection. Requests can be sent to the coordinator of the Data Governance Committee using the following email: dgc@kemri-wellcome.org.

Acknowledgments

We wish to thank all our study participants for taking time, voluntarily, to participate in this study. We would also like to thank Katana Ngombo, Richard Karisa, Beatrice Kabunda, Judith Tumaini Dzombo and Khamisi Katana for their role in data collection. The authors would also like to thank the Director of Kenya Medical Research Institute (KEMRI) for the permission to publish this work.

For peer review only - http://bmjopen.bmj.com/site/about/guidelines.xhtml

References

1. Panos, G., et al., *Mortality and morbidity of HIV infected patients receiving HAART: a cohort study.* Current HIV Research, 2008. **6**(3): p. 257-260.
2. Johnson, L.F., et al., *Life expectancies of South African adults starting antiretroviral treatment: collaborative analysis of cohort studies.* PLoS medicine, 2013. **10**(4): p. e1001418.
3. Nsanzimana, S., et al., *Life expectancy among HIV-positive patients in Rwanda: a retrospective observational cohort study.* The Lancet Global Health, 2015. **3**(3): p. e169-e177.
4. Samji, H., et al., *Closing the gap: increases in life expectancy among treated HIV-positive individuals in the United States and Canada.* PloS one, 2013. **8**(12): p. e81355.
5. Nakagawa, F., M. May, and A. Phillips, *Life expectancy living with HIV: recent estimates and future implications.* Curr Opin Infect Dis, 2013. **26**(1): p. 17-25.
6. Watkins, C.C. and G.J. Treisman, *Cognitive impairment in patients with AIDS – prevalence and severity.* HIV/AIDS (Auckland, N.Z.), 2015. **7**: p. 35-47.
7. NIMH. *HIV/AIDS and Mental Health*. National Institute of Mental Health (NIMH), 2016. [23rd Jan 2018]; Available from: https://www.nimh.nih.gov/health/topics/hiv-aids/index.shtml.
8. McArthur, J.C., et al., *Human immunodeficiency virus-associated dementia: an evolving disease.* Journal of neurovirology, 2003. **9**(2): p. 205-221.
9. Saylor, D., et al., *HIV-associated neurocognitive disorder--pathogenesis and prospects for treatment.* Nat Rev Neurol, 2016. **12**(4): p. 234-48.
10. Heaton, R.K., et al., *HIV-associated neurocognitive disorders persist in the era of potent antiretroviral therapy: CHARTER Study.* Neurology, 2010. **75**(23): p. 2087-2096.
11. Ancuta, P., et al., *Microbial translocation is associated with increased monocyte activation and dementia in AIDS patients.* PloS one, 2008. **3**(6): p. e2516.
12. Habib, A.G., et al., *Neurocognitive impairment in HIV-1-infected adults in Sub-Saharan Africa: a systematic review and meta-analysis.* International Journal of Infectious Diseases, 2013. **17**(10): p. e820-e831.
13. Grant, I., *Neurocognitive disturbances in HIV.* International review of psychiatry, 2008. **20**(1): p. 33-47.

For peer review only - http://bmjopen.bmj.com/site/about/guidelines.xhtml

14. Heaton, R.K., et al., *HIV-associated neurocognitive disorders before and during the era of combination antiretroviral therapy: differences in rates, nature, and predictors.* Journal of neurovirology, 2011. **17**(1): p. 3-16.

15. Ogunrin, O. and F. Odiase, *Motor speed and reaction time in HIV/AIDS patients: a case-control study.* African journal of AIDS research, 2006. **5**(3): p. 217-220.

16. Simioni, S., et al., *Cognitive dysfunction in HIV patients despite long-standing suppression of viremia.* Aids, 2010. **24**(9): p. 1243-50.

17. Vivithanaporn, P., et al., *Neurologic disease burden in treated HIV/AIDS predicts survival A population-based study.* Neurology, 2010. **75**(13): p. 1150-1158.

18. Hinkin, C., et al., *Medication adherence among HIV+ adults effects of cognitive dysfunction and regimen complexity.* Neurology, 2002. **59**(12): p. 1944-1950.

19. Tozzi, V., et al., *Neurocognitive impairment influences quality of life in HIV-infected patients receiving HAART.* International journal of STD & AIDS, 2004. **15**(4): p. 254-259.

20. Heaton, R.K., et al., *The impact of HIV-associated neuropsychological impairment on everyday functioning.* Journal of the international neuropsychological society, 2004. **10**(3): p. 317-331.

21. Brandt, R., *The mental health of people living with HIV/AIDS in Africa: a systematic review.* African Journal of AIDS Research, 2009. **8**(2): p. 123-133.

22. Reis, R.K., et al., *Symptoms of depression and quality of life of people living with HIV/AIDS.* Revista latino-americana de enfermagem, 2011. **19**(4): p. 874-881.

23. Ciesla, J.A. and J.E. Roberts, *Meta-analysis of the relationship between HIV infection and risk for depressive disorders.* American Journal of Psychiatry, 2001. **158**(5): p. 725-730.

24. GIP. *Double stigma, double challenge: Mental health and HIV/AIDS in Central and Eastern Europe and the Newly Independent States. Global Inititative on Psychiatry (GIP)*, 2006. [15th January 2018]; Available from: http://www.bibalex.org/Search4Dev/files/417303/362887.pdf.

25. Singer, E.J. and A.D. Thames, *Neurobehavioral Manifestations of HIV/AIDS: Diagnosis and Treatment.* Neurologic clinics, 2016. **34**(1): p. 33-53.

26. Stutterheim, S.E., et al., *HIV-related stigma and psychological distress: the harmful effects of specific stigma manifestations in various social settings.* Aids, 2009. **23**(17): p. 2353-7.

For peer review only - http://bmjopen.bmj.com/site/about/guidelines.xhtml

27. Gonzalez, J.S., et al., *Depression and HIV/AIDS treatment nonadherence: a review and meta-analysis.* J Acquir Immune Defic Syndr, 2011. **58**(2): p. 181-7.
28. Chan, L.G., N. Kandiah, and A. Chua, *HIV-associated neurocognitive disorders (HAND) in a South Asian population-contextual application of the 2007 criteria.* BMJ open, 2012. **2**(1): p. e000662.
29. Yakasai, A.M., et al., *Prevalence and correlates of HIV-associated neurocognitive disorders (HAND) in Northwestern Nigeria.* Neurology research international, 2015. **2015**.
30. NACC. *Kenya HIV County Profiles - 2016*. National AIDS Control Council (NACC), 2016. [20 July 2017]; 49-52]. Available from: http://nacc.or.ke/wp-content/uploads/2016/12/Kenya-HIV-County-Profiles-2016.pdf.
31. Robertson, K.R., et al., *Pattern of neuropsychological performance among HIV positive patients in Uganda.* BMC Neurology, 2007. **7**(1): p. 8.
32. Sunmonu, T.A., et al., *Intellectual impairment in patients with newly diagnosed HIV infection in southwestern Nigeria.* BioMed research international, 2015. **2015**.
33. Scott, J.A.G., et al., *Profile: the Kilifi health and demographic surveillance system (KHDSS).* International journal of epidemiology, 2012. **41**(3): p. 650-657.
34. Von Elm, E., et al., *The Strengthening the Reporting of Observational Studies in Epidemiology (STROBE) statement: guidelines for reporting observational studies.* PLoS medicine, 2007. **4**(10): p. e296.
35. Abubakar, A., et al., *Socioeconomic status, anthropometric status, and psychomotor development of Kenyan children from resource-limited settings: a path-analytic study.* Early human development, 2008. **84**(9): p. 613-621.
36. Abubakar, A., et al., *Cumulative Psychosocial Risk is a Salient Predictor of Depressive Symptoms among Vertically HIV-Infected and HIV-Affected Adolescents at the Kenyan Coast.* Annals of global health, 2017.
37. Bech, P., et al., *The sensitivity and specificity of the Major Depression Inventory, using the Present State Examination as the index of diagnostic validity.* Journal of affective disorders, 2001. **66**(2): p. 159-164.
38. Olsen, L., et al., *The internal and external validity of the Major Depression Inventory in measuring severity of depressive states.* Psychological medicine, 2003. **33**(2): p. 351-356.
39. Otiende, M., et al., *Psychometric evaluation of the Major Depression Inventory among young people living in Coastal Kenya.* Wellcome Open Research, 2017. **2**.

For peer review only - http://bmjopen.bmj.com/site/about/guidelines.xhtml

40. Raven, J.C., J. Court, and J. Raven, *Manual for Raven's Progressive Matrices and Vocabulary Scales. The Coloured Progressive Matrices. Oxford Psychologist Press: London.* 1977.

41. Taylor, H.G., et al., *Postirradiation treatment outcomes for children with acute lymphocytic leukemia: Clarification of risks.* Journal of pediatric psychology, 1987. **12**(3): p. 395-411.

42. Wechsler, D., *Wechsler Adult Intelligence Scale–Revised manual. The Psychological Corporation: New York, NY.* 1981.

43. Riddle, T. and J. Suhr, *Extension of the contingency naming test to adult assessment: Psychometric analysis in a college student sample.* The Clinical Neuropsychologist, 2012. **26**(4): p. 609-625.

44. Hays, R.D. and L.S. Morales, *The RAND-36 measure of health-related quality of life.* Annals of medicine, 2001. **33**(5): p. 350-357.

45. Frempong-Ainguah, F. and A. Hill, *Reliability, validity and responsiveness of the short form-36 health survey: Findings from the women's health study of Accra, Ghana.* Revue quetelet/quetelet journal, 2017. **2**(2).

46. StataCorp LP, *Stata Statistical Software: Release 14. College Station, TX: StataCorp LP.* 2015.

47. Johnson, T.R., *Violation of the homogeneity of regression slopes assumption in ANCOVA for two-group pre-post designs: Tutorial on a modified Johnson-Neyman procedure.* Tutorials in quantitative methods for psychology 2016. **12**(3): p. 253-263.

48. Ngandu, T., et al., *Education and dementia: what lies behind the association?* Neurology, 2007. **69**(14): p. 1442-50.

49. Deutsch, R., et al., *AIDS-associated mild neurocognitive impairment is delayed in the era of highly active antiretroviral therapy.* Aids, 2001. **15**(14): p. 1898-1899.

50. Sacktor, N., et al., *Combination antiretroviral therapy improves psychomotor speed performance in HIV-seropositive homosexual men.* Neurology, 1999. **52**(8): p. 1640-1640.

51. Peltzer, K., et al., *Depression and Social Functioning among HIV-infected and Uninfected Persons in South Africa.* AIDS care, 2015. **27**(1): p. 41-46.

52. Chikezie, U., et al., *Depression among people living with human immunodeficiency virus infection/acquired immunodeficiency syndrome in Benin City, Nigeria: A comparative study.* Nigerian journal of clinical practice, 2013. **16**(2): p. 238-242.

For peer review only

For peer review only - http://bmjopen.bmj.com/site/about/guidelines.xhtml

53. Araya, R., et al., *Education and income: which is more important for mental health?* Journal of Epidemiology & Community Health, 2003. **57**(7): p. 501-505.

54. Yimam, K., Y. Kebede, and T. Azale, *Prevalence of common mental disorders and associated factors among adults in Kombolcha Town, Northeast Ethiopia.* Journal of Depression and Anxiety 2014. **1**: p. 2167-1044.

55. Charles, B., et al., *Association between stigma, depression and quality of life of people living with HIV/AIDS (PLHA) in South India – a community based cross sectional study.* BMC Public Health, 2012. **12**: p. 463-463.

For peer review only

For peer review only - http://bmjopen.bmj.com/site/about/guidelines.xhtml

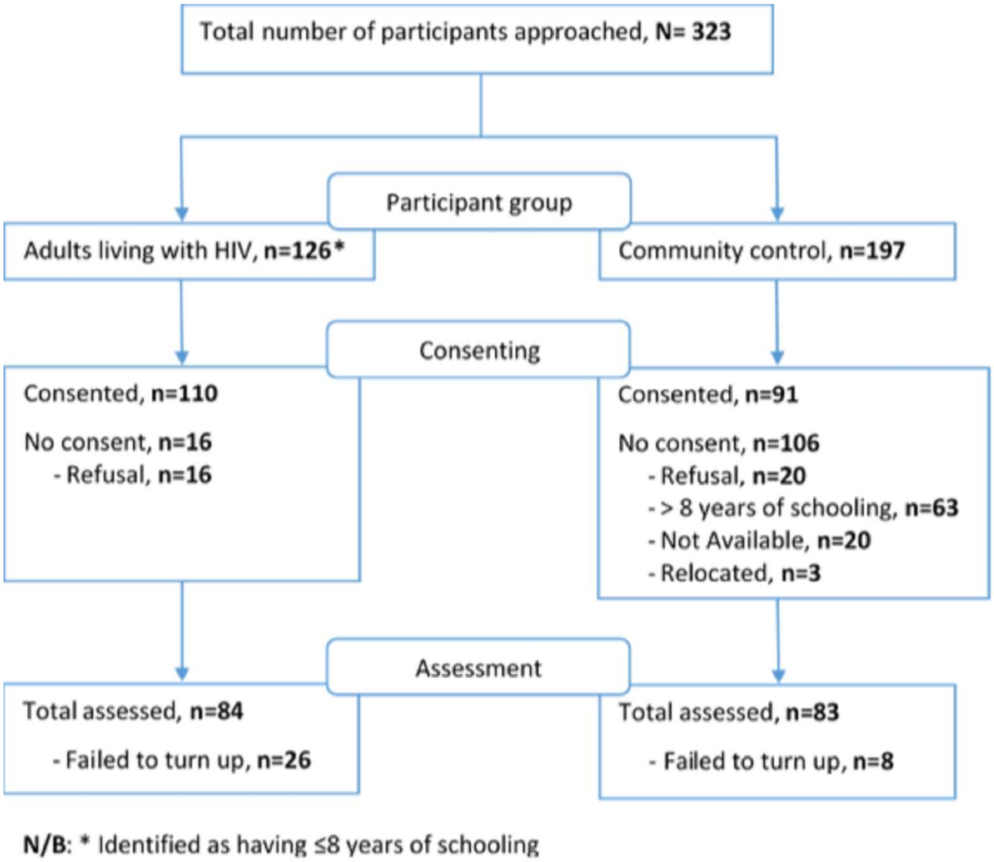

Figure 1 Flowchart of participant recruitment

114x99mm (300 x 300 DPI)

STROBE 2007 (v4) Statement—Checklist of items that should be included in reports of *cross-sectional studies*

Section/Topic	Item #	Recommendation	Reported on page #
**Title and abstract**	1	(*a*) Indicate the study's design with a commonly used term in the title or the abstract	1
		(*b*) Provide in the abstract an informative and balanced summary of what was done and what was found	2
**Introduction**			
Background/rationale	2	Explain the scientific background and rationale for the investigation being reported	4-5
Objectives	3	State specific objectives, including any prespecified hypotheses	5
**Methods**			
Study design	4	Present key elements of study design early in the paper	5
Setting	5	Describe the setting, locations, and relevant dates, including periods of recruitment, exposure, follow-up, and data collection	5
Participants	6	(*a*) Give the eligibility criteria, and the sources and methods of selection of participants	6
Variables	7	Clearly define all outcomes, exposures, predictors, potential confounders, and effect modifiers. Give diagnostic criteria, if applicable	12 (Table 3)
Data sources/ measurement	8*	For each variable of interest, give sources of data and details of methods of assessment (measurement). Describe comparability of assessment methods if there is more than one group	7-8
Bias	9	Describe any efforts to address potential sources of bias	7
Study size	10	Explain how the study size was arrived at	6
Quantitative variables	11	Explain how quantitative variables were handled in the analyses. If applicable, describe which groupings were chosen and why	9/10
Statistical methods	12	(*a*) Describe all statistical methods, including those used to control for confounding	9
		(*b*) Describe any methods used to examine subgroups and interactions	N/A
		(*c*) Explain how missing data were addressed	Pairwise deletion using statistical methods (page 9)
		(*d*) If applicable, describe analytical methods taking account of sampling strategy	9

For peer review only

		(*e*) Describe any sensitivity analyses	N/A
**Results**			
Participants	13*	(a) Report numbers of individuals at each stage of study—eg numbers potentially eligible, examined for eligibility, confirmed eligible, included in the study, completing follow-up, and analysed	Figure 1
		(b) Give reasons for non-participation at each stage	Figure 1
		(c) Consider use of a flow diagram	Figure 1
Descriptive data	14*	(a) Give characteristics of study participants (eg demographic, clinical, social) and information on exposures and potential confounders	10 (Table 1)
		(b) Indicate number of participants with missing data for each variable of interest	10 (Table 1)
Outcome data	15*	Report numbers of outcome events or summary measures	10-11
Main results	16	(*a*) Give unadjusted estimates and, if applicable, confounder-adjusted estimates and their precision (eg, 95% confidence interval). Make clear which confounders were adjusted for and why they were included	11 -12
		(*b*) Report category boundaries when continuous variables were categorized	N/A
		(*c*) If relevant, consider translating estimates of relative risk into absolute risk for a meaningful time period	N/A
Other analyses	17	Report other analyses done—eg analyses of subgroups and interactions, and sensitivity analyses	N/A
**Discussion**			
Key results	18	Summarise key results with reference to study objectives	13-14
Limitations	19	Discuss limitations of the study, taking into account sources of potential bias or imprecision. Discuss both direction and magnitude of any potential bias	14/15
Interpretation	20	Give a cautious overall interpretation of results considering objectives, limitations, multiplicity of analyses, results from similar studies, and other relevant evidence	15
Generalisability	21	Discuss the generalisability (external validity) of the study results	14/15
**Other information**			
Funding	22	Give the source of funding and the role of the funders for the present study and, if applicable, for the original study on which the present article is based	16

*Give information separately for cases and controls in case-control studies and, if applicable, for exposed and unexposed groups in cohort and cross-sectional studies.

Note: An Explanation and Elaboration article discusses each checklist item and gives methodological background and published examples of transparent reporting. The STROBE checklist is best used in conjunction with this article (freely available on the Web sites of PLoS Medicine at http://www.plosmedicine.org/, Annals of Internal Medicine at http://www.annals.org/, and Epidemiology at http://www.epidem.com/). Information on the STROBE Initiative is available at www.strobe-statement.org.

For peer review only
